# Copy number variation in *CEP57L1* predisposes to congenital absence of bilateral ACL and PCL ligaments

**DOI:** 10.1186/s40246-015-0053-z

**Published:** 2015-11-11

**Authors:** Yichuan Liu, Yun Li, Michael E. March, Nguyen Kenny, Kexiang Xu, Fengxiang Wang, Yiran Guo, Brendan Keating, Joseph Glessner, Jiankang Li, Theodore J. Ganley, Jianguo Zhang, Matthew A. Deardorff, Xun Xu, Hakon Hakonarson

**Affiliations:** Center for Applied Genomics, The Children’s Hospital of Philadelphia, 1014H, 3615 Civic Center Blvd, Abramson Building, Philadelphia, PA 19104 USA; Beijing Genomics Institute, Shenzhen, China; Center for Sports Medicine and Performance, The Children’s Hospital of Philadelphia, Philadelphia, PA USA; Individualized Medical Genetics Center, The Children’s Hospital of Philadelphia, Philadelphia, PA USA

**Keywords:** Copy number variation, Rare disease, Whole exome sequencing

## Abstract

**Background:**

Absence of the anterior (ACL) or posterior cruciate ligament (PCL) are rare congenital malformations that result in knee joint instability, with a prevalence of 1.7 per 100,000 live births and can be associated with other lower-limb abnormalities such as ACL agnesia and absence of the menisci of the knee. While a few cases of absence of ACL/PCL are reported in the literature, a number of large familial case series of related conditions such as ACL agnesia suggest a potential underlying monogenic etiology. We performed whole exome sequencing of a family with two individuals affected by ACL/PCL.

**Results:**

We identified copy number variation (CNV) deletion impacting the exon sequences of *CEP57L1*, present in the affected mother and her affected daughter based on the exome sequencing data. The deletion was validated using quantitative PCR (qPCR), and the gene was confirmed to be expressed in ACL ligament tissue. Interestingly, we detected reduced expression of *CEP57L1* in Epstein–Barr virus (EBV) cells from the two patients in comparison with healthy controls. Evaluation of 3D protein structure showed that the helix-binding sites of the protein remain intact with the deletion, but other functional binding sites related to microtubule attachment are missing. The specificity of the CNV deletion was confirmed by showing that it was absent in ~700 exome sequencing samples as well as in the database of genomic variations (DGV), a database containing large numbers of annotated CNVs from previous scientific reports.

**Conclusions:**

We identified a novel CNV deletion that was inherited through an autosomal dominant transmission from an affected mother to her affected daughter, both of whom suffered from the absence of the anterior and posterior cruciate ligaments of the knees.

**Electronic supplementary material:**

The online version of this article (doi:10.1186/s40246-015-0053-z) contains supplementary material, which is available to authorized users.

## Introduction

Congenital absence of the anterior (ACL)/posterior cruciate ligaments (PCL) is an extremely unusual condition with a prevalence of 1.7 per 100,000 live births and was first reported in 1956 in a radiographic study of the knee [1–3]. The symptom of this disease usually associated with serious malformation or dislocation of the knees. ACL is the most common disorder, and it can be associated with hypoplasia or total PCL.

There is little knowledge of the genetics information related to this disease, but a previous study in a large number of families suggested autosomal dominant inheritance of ACL [4]. In this study, we uncovered two individuals (mother and her daughter) in a large cohort of subjects with rare diseases, and we confirmed the underlying sequence copy number variations (CNVs) that predisposes to this disease.

## Methods

### Data selections

Among 662 individuals from 212 families with Mendelian disease phenotypes, there was one family with two affected patients, including a mother (ID#4779933454) and her 13-year-old daughter (ID#1993197298), suffering from an absence of bilateral ACL and PCL ligaments. Their history was notable for joint instability and difficulty with ambulation. Absence of ACL and PCL was confirmed on MRI and by arthroscopy. The proband (daughter) has an unaffected younger sister and unaffected father, for both of whom DNA was not obtainable. However, the pattern of inheritance suggests it is autosomal dominant [4]; no other extended family members have a history of joint or lower-limb disorders.

### Exome capture and sequencing

Following the manufacturer’s protocols, we used Agilent SureSelect Human All Exon Kit (in solution) (Agilent Technologies, Santa Clara, CA, USA) to perform exome capture for the family included in this study. We used Covaris AFA (Covaris, Woburn, MA, USA) to randomly fragment genomic DNA samples with an average size of 150–200 bp and then attached adapters to both ends. We applied AgencourtAMPure SPRI beads (Beckman Coulter, Brea CA, USA) to purify the adapter-ligated templates with an insert size of around 250 bp. We employed ligation-mediated polymerase chain reaction (LM-PCR) and SureSelect Biotinylated RNA Library (BAITS) (Agilent Technologies, Santa Clara, CA, USA) to amplify, purify, and hybridize DNA for enrichment. We then washed out non-hybridized fragments after 24 h and estimated the magnitude of enrichment through capture LM-PCR products by using an Agilent 2100 Bioanalyzer (Agilent Technologies, Santa Clara, CA, USA). The Hiseq2000 platform (Illumina, San Diego, CA, USA) started paired-end sequencing with read lengths of 90 bp. We applied Illumina base-calling software V.1.7 at its default parameters to process raw image files for base-calling.

### Exome data analysis

We used two independent analysis pipelines to perform alignment, variant calling, and annotation. Pipeline (1). Sequencing reads were aligned to the human reference genome (UCSC hg19) with Burrows–Wheeler Aligner (BWA, version 0.6.2) [5]. Optical and PCR duplicates were marked and removed with Picard (version 1.73). Local realignment of reads containing indel sites and base quality score recalibration (BQSR) were performed with the Genome Analysis Tool Kit (GATK, version 2.3) [6]. Single nucleotide variation (SNV) and small indels were called with GATK UnifiedGenotyper. Variants were marked as potential sequencing artifacts if the filters on the following annotations were evaluated to be true: (1) for SNVs, “DP < 10,” “QD < 2.0,” “MQ < 40.0,” “FS > 60.0,” “HaplotypeScore > 13.0,” “MQRankSum < −12.5,” “ReadPosRankSum < −8.0,” (2) for small indels, “DP < 10,” “QD < 2.0,” “ReadPosRankSum < −20.0,” “InbreedingCoeff < −0.8,” “FS > 200.0.” The kinship coefficient was calculated for each sample using KING [7] to confirm reported relationships and identify cryptic relationships among samples. ANNOVAR [8] and SnpEff (version 2.0.5) [9] were used for annotating variants. Human gene mutation database (HGMD) [10] was used for annotating known genes and mutations for human inherited diseases. Prediction scores from SIFT [11], Polyphen2 [12], LRT [13], and MutationTaster [14] along with conservation scores PhyloP [15] and GERP++ [16] for every potential nonsynonymous SNV in the human genome were retrieved from dbNSFP (database for nonsynonymous SNPs’ functional predictions) [17].

Pipeline (2). Fastq files were aligned to the human reference genome (UCSC hg19) with the short oligonucleotide analysis package (SOAP, version 2.2.1) [18]. SOAPsnp (version 1.05) [19] was used for single nucleotide variant (SNV) detection and GATK for small insertion-deletion (indel) detection, followed by BGI’s self-developed programs to perform variant functional annotation.

### SNV detection, variant filtering, and prioritization

A series of common filtering criteria were applied to all candidate variants in both pipelines as described below. First we excluded variants that were (1) not within an exon, a predicted splice site, or an UTR; (2) synonymous changes, and (3) with minor allele frequency (MAF) >0.5 % in either the 1000 Genomes Project (http://www.1000genomes.org/), the Exome Sequencing Project (ESP6500; http://evs.gs.washington.edu/EVS/), or our internal exome datasets. Variants near splicing donor/recipient sites and frameshift indels were given particular attention as they could cause pathogenic changes such as exon-skipping, premature truncation, stop loss, or stop gain, as well as frameshifts.

We considered a number of possible genetic models in variant prioritization, which included filtering out of variants based on (1) evolutionary conservation, i.e., variants of PhyloP [15] value <0.95 were considered to be in non-conserved regions thus discarded; (2) prediction of pathogenicity by both PolyPhen [12] and SIFT [15]; and (3) biological and clinical relevance of identified variants with emphasis on pathways and interaction networks of known genes and/or proteins pertinent to the disease.

Given that congenital absence of ACP/PCL is thought to be a dominantly inherited syndrome, we used a de novo dominant model of Mendelian inheritance in our variant prioritization and filtering methodology [20]. Specifically, we focused on variants found to be heterozygous and strongly deleterious in the proband and affected parents. SNVs and indels were selected as potential pathogenic variants if they met all the following criteria: (1) heterozygous in proband and in the affect maternal sample; (2) not previously described or rare (MAF < 0.5 %) in a control cohort of more than 9000 control individuals (1000 genomes project, April 2012 release; 6503 exomes from NHLBI GO Exome Sequencing Project (ESP6500SI) and 1200 in-house whole-exomes; (3) nonsynonymous, or splice acceptor and donor site SNVs, or frame shift coding indels (NS/SS/I); (4) predicted to be deleterious by at least three prediction methods, i.e., SIFT, PolyPhen2, MutationTaster, and LRT; and (5) conserved PhyloP score and GERP++ score >2.0. Variants were also analyzed using the ingenuity variant analysis web-based application. As both affected were female, we also included in our analysis all variants on the *chr X*, considering both heterozygous dominant and hemizygous dominant scenarios. SNVs and indels fulfilling these criteria were retained as potential pathogenic variants if they also then met all the criteria 2–5 (above).

### CNV analysis for exon sequencing

CNV analysis was performed by using the standard exome hidden Markov model (XHMM) pipeline that consisting of six steps [21]. The depth of coverage for all targets and all 662 samples used for case/control comparison for this study was performed using GATK. (2) Target regions with extreme GC content (<10 % or >90 %) and low complexity regions are filtered out from further analysis. (3) PCA normalization of read depth is performed for all samples to remove inherent biases in sample preparation and sequencing. (4) Samples with extreme variability in normalized read depth are removed. (5) Per-sample CNV Detection with a HMM is performed. (6) Quality metrics are assigned to all samples for discovered CNVs.

The raw outputs of XHMM were further processed by selecting the region with “Y” annotation, which indicates the CNV region existed in the proband. The selected regions were then compared with the clinical phenotypes; the regions were further selected if the clinical phenotype matches the existence of the CNV. In other words, if the individual in this family has the disease, CNV presence in the individual is required and CNV absence if the individual is healthy.

### Computational validation for CNVs

Since CNVs are common across the human genome, it is important to determine CNV specificity during the CNV validation processes. In this study, multiple validations were applied in order to increase the confidence of the CNV hits. First, we checked whether the CNV hit is presented in 274 healthy individuals; second, we checked whether the CNV hit existed in any of the 147 families with different monogenic diseases, including 388 individuals also called at the same time for this study (Additional file 1: Table S1); finally, we checked whether the CNV hit is present in the database of genomic variations (DGV), which contains annotated CNVs from previous scientific reports [22]. If a CNV hit presented only in the patients but not in other populations and had not been reported in other previous studies, it is more confident to conclude that the CNV may be relevant or potentially associated with the target disease.

### qPCR validation for CNVs

CNV Validation by quantitative PCR (qPCR) with the Universal Probe Library (UPL): CNV validation by qPCR was performed roughly as described previously [23, 24]. UPL probes (Roche, Indianapolis, IN) and corresponding primers were selected using the ProbeFinder v2.49 software (Roche, Indianapolis, IN). Probes used and primer sequences are provided in Table 2. All primers were tested to ensure that they generated a PCR product of the correct approximate size. All primer probe combinations were also tested on a dilution series of control DNA to determine PCR efficiencies. All assays achieved PCR efficiency between 95 and 105 %. Quantitative PCR was performed on an ABI Prism™ 7900HT Sequence Detection System (Applied Biosystems, Foster City, CA). Each sample reaction was performed in triplicate, in 10 ul of reaction mixture containing 10 ng genomic DNA, 100 nM of the UPL probe, 400 nM of each PCR primer, and 1× TaqMan Gene Expression Master Mix containing UDG and ROX (Life Technologies, Carlsbad, CA). Male and female genomic DNAs (Promega, Madison, WI) were included in the analysis as positive controls for subjects with expected normal copy number. Data were evaluated using the Sequence Detection Software v2.4 (Applied Biosystems, Foster City, CA). Data was further analyzed by the ∆ΔC_T_ method. The geometric mean of the C_T_ values for the two control sequences (GAPDH and SNCA) was calculated and used as the reference value for ∆ΔC_T_ calculations. The Promega female DNA sample was considered the reference 2N sample for ∆ΔC_T_ calculations. Hemizygous deletions were determined when the relative copy number value for a specific sample normalized to the reference sample was less than 0.75.

### EBV cells

Epstein–Barr virus (EBV) cells were developed from the proband and affected family member (mother), together with cells from four healthy controls described in next section. The EBV-transformed cell lines from the six individuals were cultured into exponential growth, and 5 million cells were harvested for RNA isolation. Thus, two of the EBV cell lines were from the individuals harboring the hemizygous deletion in *CEP57L1*, whereas the other four cell lines were from randomly selected individuals from the CAG bio-repository representing normal *CEP57L1* genotypes and expression. Expression of *G6PD* was observed to be variable between EBV cell lines, so for the analysis of expression from the EBV cells, only *ACTB* and *B2M* were used as controls. In addition, for analysis of expression in EBV-transformed cells, a sample from an individual not carrying the predicted deletion was used as the control sample for calculations.

### qPCR validation for EBV cells and ligament tissues from knee surgery

Samples of anterior cruciate ligament (ACL) tissue were isolated from four individuals undergoing ACL surgeries. Samples were stored at −80C prior to RNA isolation. RNA was isolated from homogenized tissue samples or cell lysates using the RNeasy Mini Kit (Qiagen, Venlo, Netherlands) with optional on-column DNAse digestion, following the manufacturer’s instructions. ACL samples (approximately 200 mg tissue) were homogenized in buffer RLT using a PowerGen 125 Homogenizer (Fisher Scientific, Waltham, MA). EBV-transformed cell lines (5 × 10^6^ cells) were homogenized in buffer RLT using QiaShredder columns (Qiagen).

Isolated RNA was converted to cDNA using the High Capacity RNA-to-cDNA Kit, following manufacturer’s protocol. Reactions involving RNA from ACL tissue samples utilized 300 ng of total RNA in a 20 μl l reaction volume, while reactions with RNA from EBV cells were performed using 2 μl g of RNA in 20 μl l reactions. Additionally, control reactions were performed for each sample that excluded the reverse transcriptase. Gene expression analysis was performed by qPCR with the reverse transcribed cDNA using the Universal Probe Library system (Roche, Indianapolis, IN). UPL probes and corresponding primers were selected using the ProbeFinder v2.49 software (Roche). Probes used and primer sequences are provided in Table 1. Quantitative PCR was performed on an ABI ViiA 7 Real-Time PCR System (Applied Biosystems, Foster City, CA). Each sample reaction was performed in triplicate, in 10 ul of reaction mixture containing cDNA, 100 nM of the UPL probe, 400 nM of each PCR primer and 1× TaqMan Gene Expression Master Mix containing UDG and ROX (Life Technologies, Carlsbad, CA). Reactions involving ACL tissue RNA contained cDNA corresponding to 5 ng of the starting RNA. Reactions with the EBV cell line RNA contained cDNA corresponding to 80 ng of starting RNA. Quantitative PCR was performed both on the converted cDNA and the corresponding control, no reverse transcriptase reactions in order to control for contamination of RNA with genomic DNA. Data was analyzed by the ∆ΔC_T_ method. The geometric mean of the C_T_ values for the control genes (beta-actin/*ACTB*, glucose-6-phosphate dehydrogenase*/G6PD*, and beta-2-microglobulin/*B2M*) was calculated and used as the reference value for ∆ΔC_T_ calculations. For analysis of ACL expression, a sample was chosen at random to use as the normalization control.

**Table 1:** Assay design for qRT-PCR analysis of gene expression in ACL tissue or EBV LCLs

Gene symbol—probe name	Gene	Amplicon position	UPL probe #	Left primer^a^	Right primer^b^
*CEP57L1*—exons 4-5	Centrosomal protein 57 kDa-like 1	Spans exons 4 and 5 of human CEP57L1 transcript variant 1	78	agcccaaatagccaagctc	tctctggaaagaatgttcaggtt
*CEP57L1*—exons 5-6	Centrosomal protein 57 kDa-like 1	Spans exons 5 and 6 of human CEP57L1 transcript variant 1	17	caaatgagagaaatctggcaca	acgagactgggctgagctt
*CEP57L1—*exon 1	Centrosomal protein 57 kDa-like 1	Contained within exon 1 of human CEP57L1 transcript variant 1	80	ctaagcttgcgccctgag	cgaagtctcagtgtacttctacgtct
*ACTB*	Beta-actin	Spans exons 4 and 5 of human ACTB transcript	11	attggcaatgagcggttc	cgtggatgccacaggact
*B2M*	Beta-2-microglobulin	Spans exons 1 and 2 of human B2M transcript	42	ttctggcctggaggctatc	tcaggaaatttgactttccattc
*G6PD*	Glucose-6-phosphate dehydrogenase	Spans exons 4 and 5 of human G6PD transcript	82	Gcaaacagagtgagcccttc	gagttgcgggcaaagaagt

^a^All primers are listed 5′ to 3′

### CEP57L1 modeling

Centrosomal protein 57 kDa-like protein 1 (*CEP57L1*) is a protein-coding gene that aids in (1) microtubule binding through filaments composed of tubulin monomers, (2) identical protein binding, and (3) γ-tublulin binding with the microtubule constituent protein of the same name. Each function interacts selectively and non-covalently. A three-dimensional (3D) model of *CEP57L1* was rendered using Phyre2 [25]. The structure created by Phyre2 was chosen due its high confidence, and model quality estimations were done for structural validation [26–28]. The sequence of the wild-type (wt) *CEP57L1* was obtained from the UniProt database (accession number Q7IYX8). Models and quality estimations were done through the protein model portal (PMP), which is part of the Structural Biology Knowledgebase (SBKB, sbkb.org), Protein Structure Initiative (PSI), and Nature Publishing Group (NPG). PEP-FOLD was used to determine the structure of residues 1 to 18, which resulted in an α-helical motif (Fig. 3c) [26]. A Monte Carlo simulation of CEP57L1 was performed using PELE from the Barcelona Supercomputing Center (BSC) [29]. The simulation generated 67 low-energy conformations out of 100 steps in less than 24 h using one central processing unit (CPU) as requested by the BSC for searching protein local motion.

## Results

### Discoveries and computational validation of rare variants underlying ACL/PCL

**Fig. 1:**
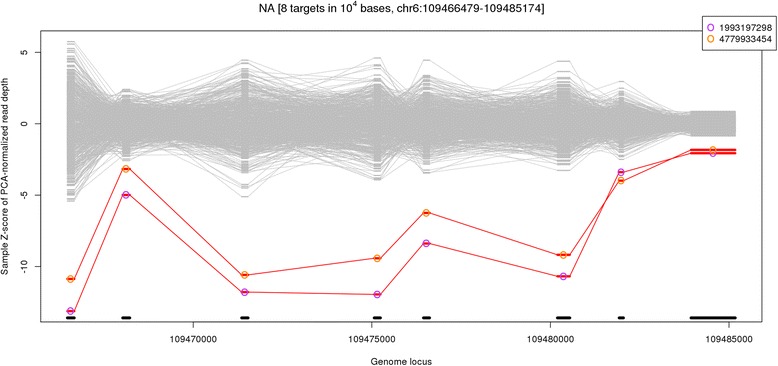
XHMM plots for copy number variations (CNV): CNV deletion region for chr6:109466479-109485174. *x-axis* represents the genome locus, and y-axis is the computed Z-score of PCA normalized read depth; *positive values* indicate duplication and *negative values* indicate deletion; target individuals are highlighted with *color* while *gray lines* are the control individuals from 663 population pool

We used WES in search for rare causative variants explaining ACL/PCL. We ran our WES data through our discovery pipeline as previously described [27], and we did not identify any SNVs that segregated in the family that were likely to be causative of ACL/PCL. We next evaluated if there were any CNVs that could explain the disease. The major problem of CNVs discovery is the specificity of the findings due to the prevalence of CNVs across the human genome. Therefore, multiple filters have been applied to remove the potential false positive CNVs. First, the CNV should not be present in the 274 healthy individuals called at the same time. In other words, the CNV should not be present among healthy controls to explain the association with absence of ACL/PCL. The target CNV deletions occurred within the genomic region on chr6:109466479-109485174 (Fig. 1). From the XHMM plots, the region shows clearly CNV deletions in the proband and her mother and its absence in the healthy controls.

Besides the exclusions of the CNVs in healthy controls, it is important that the CNV regions are exclusive to other rare diseases, implying that the CNV should be unique to the ACL/PCL phenotypes in this instance. In order to further validate the results, we collected additional 388 individuals who have wide ranges of rare disease (147 different diagnoses). Again, the CNV region is not identified in any of those individuals. We expanded the culprit CNV region to include 50k base pairs up/downstream at the CNV locus. The region is still exclusive and different from any CNVs identified in the healthy control or the different disease groups. We additionally evaluated the CNVs through DGV, which is a genome variation database based on published CNV regions. Many of the studies reported in DGV contain very large populations with large number of CNVs [22]. No genome variations have been reported before in DGV for the culprit region, indicating that there is a novel CNV.

The computational validation enhances the specificity of the CNV regions we identified. Based on multiple filters we applied, the results show that the CNV region could be a potential variation that is responsible for the absence of ACL/PCL phenotypes.

### qPCR validation for CNVs

**Fig. 2:**
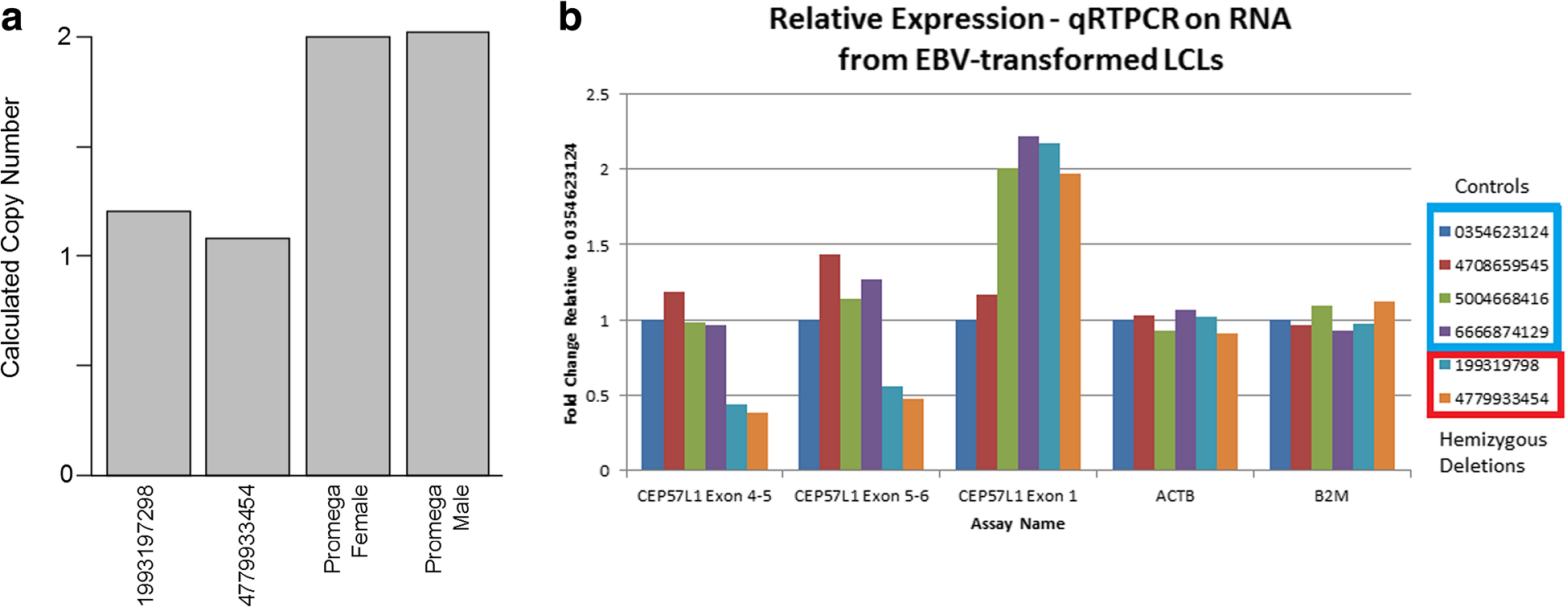
Experimental validation of the CNV: **a** qPCR results and **b** qPCR for gene expression based on EBV cells

Quantitative PCR using UPL probes was performed to determine the copy number of *CEP57L1* genomic sequences in the proband and her mother (Fig. 2a). Primer and probe combinations were designed against *CEP57L1* genomic DNA sequence, and an assay located in an intron, approximately 400 base pairs upstream of exon 7, was selected for validation. The genomic coordinates of each targeted amplicon are listed in Table 2. The tested location was shown to exist as a hemizygous deletion (1N copy number) in the two affected individuals.

**Table 2:** qPCR validation primer information for EBV cells genomic DNA

Gene name	Gene	Amplicon position^a^	UPL probe #	Left primer^b^	Right primer^b^
*CEP57L1*	Centrosomal protein 57 kDa-like 1	Amplicon located in intronic genomic DNA between exons 6 and 7 (chr6:109474636-109474742) chr6:109474636-109474742	75	gaggggtcccgttatgttg	gcgtggtggctcatacttg
*GAPDH*	Glyceraldehyde 3-phosphate dehydrogenase	chr12: 6645563-6645625	10	gctgcattcgccctctta	gaggctcctccagaatatgtga
*SNCA*	Synuclein, alpha	chr4: 90743466-90743537	68	gctgagaagaccaaagagcaa	ctgggctactgctgtcacac

^a^Amplicon position as reported by UCSC genome browser (hg19) in silico PCR tool

^b^All primers are listed 5′ to 3′

### qPCR validation for CEP57L1 expression profiling in EBV cells

To determine if *CEP57L1* is expressed in anterior cruciate ligament (ACL) tissue, we extracted RNA from ACL tissue samples from four individuals who had undergone surgeries on their ACLs. These subjects were unrelated to the individuals who contained *CEP57L1* deletions. RNA from ACL tissues was converted to cDNA and subjected to qPCR using a series of assays targeting three different regions of the *CEP57L1* messenger RNA (mRNA) or one of three control mRNAs (*B2M*, *ACTB*, *G6PD*). Amplification of *CEP57L1* mRNA was observed in all four RNA samples, to variable degrees depending on the assay used. Each of the assays completely failed to amplify anything from RNA samples that had been subjected to control reverse transcriptase reactions from which the reverse transcriptase enzyme had been excluded. We conclude that *CEP57L1* is expressed in ACL tissue.

To determine if the hemizygous deletion affected expression of *CEP57L1*, we extracted RNA from cell lines generated by EBV transformation of peripheral blood mononuclear cells from the proband, her mother, and four healthy controls. RNA from the two deletion containing subjects was compared to RNA from four subjects randomly selected from the CAG bio-repository (Fig. 2b). Exon 1 of the *CEP57L1* mRNA lies outside the detected deletion so the expression of exon 1 is expected to be unaltered. Detection of *CEP57L1* mRNA with an assay targeted exon 1 showed that the t levels of expression of *CEP57L1* mRNA in the cases and controls were similar. Exons 4 through 6 are contained within the detected deletion. Using assays that detect the junction of exons 4 and 5 or the junction of exons 5 and 6 revealed relatively similar levels of expression of these more 3′ regions of the *CEP57L1* mRNA in all four of the control subjects. In comparison, both deletion subjects showed lower expression of these regions of the mRNA, at approximately 40–50 % of the control subjects. We interpret these data to indicate that the deletion subjects possess approximately half the amount of functional, full length *CEP57L1* mRNA as control subjects.

### CEP57L1 structural analyses

**Fig. 3:**
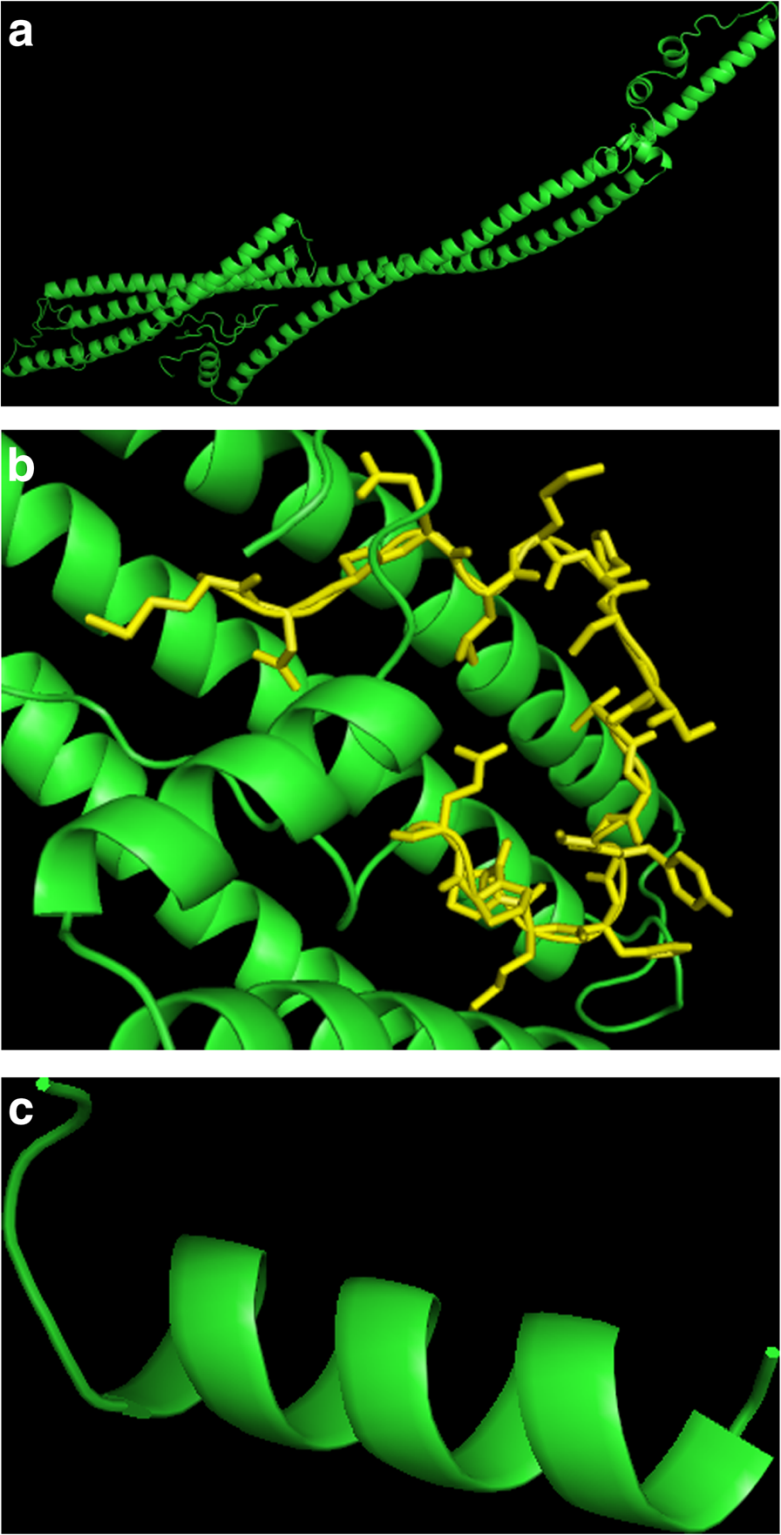
The 3D structure for *CEP57L*: **a** Phyre2 was used to create the model, which 95 % of the 460 residues were modeled at >90 % confidence using 1CII as a template, **b** Residues 1 to 18 (*yellow*) are zoomed in and is also the remaining structure—the *bottom left* disordered region in **a**—from the exon deletion and are surrounded by an α-helical-dominant protein, and **c** the result from the absence of the surrounding helices as they form a higher-ordered-α-helical structure predicted by PEP-FOLD in which intermolecular interactions may have contributed to a more disordered behavior

The 3D structure of CEP57L1 was chosen from Phyre2 due to its high confidence (Fig. 3a). Residues 1 to 18 may lack a secondary structure due to the intermolecular interactions surrounding them—specifically, the 178 residues that make up the coiled-coil domain from position 51 to 228 (Fig. 3b). Residues 1 to 18 only remain after exon deletion and was modeled using PEP-FOLD, an ab initio protein structure predictor [30]. Absence of the coiled-coil domains suggest that residues 1 to 18 may result in an α-helical structure (Fig. 3c). A Monte Carlo simulation was performed (without the presence of water) to determine if a secondary structure was generated in residues 1 to 18. As a result, they did not generate a secondary structure in the presence of a coiled-coil domain and the rest of CEP57L1 [29]. Experimental evidence confirms that the C terminus is responsible for bundling and nucleating microtubules in vivo via binding [28]. As for the N terminus, the coiled-coil domain interacts with the centrosome internal to γ-tublulin and can also mutlimerize with the N terminus of other CEP57 structures [28]. Therefore, the absence of the N- and C-termini, as well as the remaining fragment of residues 1 to 18 after exon deletion, cannot support all three functions since 96 % of CEP57L1 is absent in any interaction. All images were created using pyMOL.

## Discussion

Absence of the ACL/PCL constitutes a rare congenital malformation that results in knee joint instability with a prevalence of 1.7 per 100,000 live births and can be associated with other lower-limb abnormalities such as ACL agnesia and absence of the knee menisci. Previous studies of several families suggested that the potential inheritance pattern is under Mendelian etiology [4, 29, 30]. In this study, we focus on the family with both mother and daughter who are notable for joint instability and difficulty with ambulation. Absence of ACL and PCL were confirmed on MRI and by arthroscopy. Since other family member’s DNA was not obtainable, we applied the analysis in different molecular levels to explore the potential causation of the disease.

We performed a thorough analysis in search for SNVs that may underlie ACL/PCL. No detrimental variant was identified that could explain the disease. We next searched for CNVs to determine if any such variant with detrimental effects segregated in the family. CNVs are structural variations in the genome that lead to either deletions or duplications of chromosome regions of a variable size. CNVs have been proving to associate with susceptibility to disease, such as cancer [31], autism [24], and schizophrenia [32]. Previous research also suggests that inherited CNVs are associated with Mendelian diseases [33]. Due to potential underlying Mendelian etiology for the ACL/PCL phenotype, the genetic mechanism could be studied by CNV exploration. Exome sequencing has been shown to enhance Mendelian disease gene identification resulting in improved clinical diagnosis, more accurate genotype-phenotype correlations and new insights into the role of rare genomic variation in disease, both SNVs and CNVs. Therefore, we used WES to explore the potential of identifying SNVs or CNVs that may be associated with ACP/PCL with exome sequencing technology in this study.

Due to the high prevalence of CNVs in human genome [34], any potential pathogenic CNV needs to be shown to be absent in healthy individuals or patients with other unrelated diseases. We applied several filters to improve the confidences of our results. First, we collected a relatively large number of individuals who were healthy controls (*n* = 274) as well as 388 individuals that covered a wide range of 144 different rare diseases. By checking the CNVs across those individuals, we make sure the CNVs we identified are not present in healthy controls or in other rare disease group. To overcome the size limitation of the control set, we next examined the CNVs previously reported in the DGV database, which contains many large CNV studies in healthy populations, including over ten thousands individual. If the CNV is absent in the DGV database, it indicates that the CNV is novel and lends support that the CNV may be associated with the disease of interest, which was the case in our study of the ACL/PCL phenotype. Indeed, we identified a novel CNV deletion region on chr6:109466479-109485174, corresponding to gene *CEP57L1*, we showed the CNV segregated in the family, and we validated the deletion by qPCR.

In order to explore the impact of the CNV deletion we identified, we applied functional studies examining RNA gene expression and the 3D protein structure modeling. We performed qPCR on the corresponding EBV cells of those two individuals. The deletion occurs at region chr6:109466479-109485174, which is corresponding to 10 exons for gene *CEP57L1*. The rationale is if the CNVs have effects for clinical phenotype, they would cause alternation of expression at the gene level. The results from qPCR show that the CNV deletions possess approximately half the amount of functional, full length *CEP57L1* mRNA as control subjects indicates that the de novo CNV deletion would lead to decrease of gene expression. In the protein structure modeling, we prove that the functional sites would lose while the binding site of the protein intact, indicating that with the CNV deletion, the protein could still binding to its targets without available functions.

In conclusion, we discovered a novel CNV associated with ACL/PCL, which has high potential to be the causation of the disease. The confidence is built on the robust discovery set, the novelty of the finding, and strong supports from functional studies in gene and protein levels. In other words, in this project we applied ~700 exome sequencing data for CNV discoveries, the CNVs are validated computationally through multiple filters and database, and the existences of CNV were validated through qPCR experimentally. The corresponding gene expression in tissues were measured and compared to the healthy individuals, and the 3D protein structures were simulated to enhance the confidence of the CNV impact. While interventions at the level of *CEP57L1* may not be feasible, the role of this gene in the pathogenesis of ACL/PCLagenesis helps explain disease causality.

### Ethics statements

This study had been approved by the Children’s Hospital of Philadelphia with IRB# 4886. All the patients who participated in this project have been consented and agree to publish the results.

## Additional file

Additional file 1: Table S1. Family information, individual clinical diagnoses and race for 662 individuals. Those individuals are used as filters for CNV discoveries, including 274 healthy and 388 cases with different rare diseases.
