# Erratum to: Copy number variation in *CEP57L1* predisposes to congenital absence of bilateral ACL and PCL ligaments

**DOI:** 10.1186/s40246-016-0060-8

**Published:** 2016-01-18

**Authors:** Yichuan Liu, Yun Li, Michael E. March, Kenny Nguyen, Kexiang Xu, Fengxiang Wang, Yiran Guo, Brendan Keating, Joseph Glessner, Jiankang Li, Theodore J. Ganley, Jianguo Zhang, Matthew A. Deardorff, Xun Xu, Hakon Hakonarson

**Affiliations:** Center for Applied Genomics, The Children’s Hospital of Philadelphia, 1014H, 3615 Civic Center Blvd, Abramson Building, Philadelphia, PA 19104 USA; Beijing Genomics Institute, Shenzhen, China; Center for Sports Medicine and Performance, The Children’s Hospital of Philadelphia, Philadelphia, PA USA; Individualized Medical Genetics Center, The Children’s Hospital of Philadelphia, Philadelphia, PA USA

After publication of this article [1], the authors noticed that Kenny Nguyen’s name had been incorrectly displayed as Nguyen Kenny. The correct presentation of Kenny Nguyen’s name is included in the author list above and has also been updated in the original article [1].
